# Phenotypic Cuticle Plasticity at High Elevation: Is Microstructure and Microchemistry Related to Water Permeability?

**DOI:** 10.1111/pce.70344

**Published:** 2025-12-18

**Authors:** Giuseppe Tiloca, Othmar Buchner, Matthias Stegner, Notburga Gierlinger, Gilbert Neuner

**Affiliations:** ^1^ Institute of Biophysics University of Natural Resources and Life Sciences Vienna Austria; ^2^ Department of Botany University of Innsbruck Innsbruck Austria

**Keywords:** alpine microclimate, cuticle, irradiation exposure, leaf minimum diffusive conductance, phenolics, Raman microscopy, slope effect

## Abstract

*Kalmia procumbens* (*K. procumbens*), a ubiquitous alpine dwarf shrub, thrives at high elevations, particularly on wind‐exposed sites. Plants on contrasting north‐ and southeast‐facing slopes at ~2237 m elevation exhibit differences in leaf colour and growth, suggesting acclimative strategies. Leaves from the southeast‐facing slope, exposed to higher leaf temperatures (54°C), stronger winds (21 m ∙ s^−1^), and increased solar irradiation (2319 µMol photons m^−2^ ∙ s^−1^), developed thicker cuticles (16 µm) than leaves from north‐facing slope (11 µm). Raman imaging revealed that cutin and triterpenoids built the foundation in all cuticles on the adaxial and abaxial leaf sides sampled from the two contrasting slopes, while flavonoids accumulated mostly in the outer adaxial cuticle layer and reached the highest values at the *N‐site*. The thicker cuticle of the *S‐site* was mainly composed of cutin and triterpenoids, while the flavonoids were restricted to a thinner outer layer. Minimum diffusive conductance (*g*_min_) was lower in the *S‐site* leaves, which may be associated to their thicker cuticle. The water permeability (*g*_min_) increased exponentially with temperature in leaves from both slopes. Under heat, above 38°C, north‐facing leaves with higher flavonoid content lost increasingly more water. While the flavonoids will defend bacterial and fungal pathogens and have a vital role in enhancing plant resilience, they seem to promote higher water permeability of the cuticle of *K. procumbens*. By combining physiological, structural and chemical insights, our findings suggest that micro‐environmental factors play a significant role in driving acclimative responses in *K. procumbens*. Cuticle structure, composition and function are finely tuned to alpine microhabitats and illustrate a distinct potential for phenotypic adjustment to environmental and biotic constraints.

## Summary statement

Microenvironmental differences at high elevation drive acclimation responses in the cuticle of *Kalmia procumbens* within its phenotypic plasticity. Fine‐tuning of water permeability is achieved by structural and chemical adjustments (thickness and flavonoid content), while cutin and triterpenoids form the cuticle foundation. Altogether is key for stress survival.

## 1 Introduction

In mountainous regions, the interplay between complex topography and atmospheric conditions that change with elevation gives rise to a rich mosaic of habitats (Scherrer and Körner 2010). The orientation of slopes and their angle of exposure to sunlight create distinct microclimates, with south‐facing slopes generally experiencing warmer temperatures than north‐facing slopes in the Northern hemisphere (Bliss 1962; Moser et al. 1977; Körner 1999; Crawford 2008). These unique microclimates result in an impressive diversity of plant species and communities in close proximity (Cantlon 1953; Körner 1999; Bennie et al. 2008). The phenotypic plasticity enables individual species to express different phenotypes in response to varying environmental conditions through structural and functional responses and allows for immediate acclimation while potentially influencing long‐term adaptation processes (Pigliucci et al. 2006; Zeng 2014; Yang et al. 2020; Wang et al. 2025). An example of such processes is the species *Arabidopsis arenosa*, as in several European mountain ranges alpine ecotypes evolved from a widely distributed foothill ecotype, accompanied by parallel changes in multiple phenotypic and functional traits (Knotek et al. 2020; Bertel et al. 2022; Kaplenig et al. 2022; Bertel et al. 2025). This intraspecific plasticity seems to be a crucial factor in adapting to ongoing environmental changes (Kiełtyk 2021; Rixen et al. 2022; Chen et al. 2023).

Changes in phenotypic traits observed in response to contrasting microenvironments can be used to unravel phenotypic plasticity. An alpine species that thrives under a wide range of environmental conditions is *Kalmia procumbens* (*K. procumbens*; Larcher 1977; Moser et al. 1977). On windward sites, it forms dense, prostrate and ‘creeping’ carpets having a number of specific morphological, anatomical and physiological traits (Cernusca 1976; Moser et al. 1977; Reisigl and Keller 1987). While persistent wind exposure shapes its creeping morphology in alpine habitats, it can be found on both north‐ and south‐facing slopes, which have extremely contrasting temperatures owing to different irradiation exposures. Thus, *K. procumbens* is an excellent model for studying the functional and phenotypic plasticity of individuals growing naturally in close proximity under the influence of contrasting temperature and irradiation climates but under similar macro‐environmental conditions.

To thrive in the challenging low‐temperature environment of the mountains, plants have evolved small growth forms (Körner 1999, 2016; Neuner et al. 2000; Nagy et al. 2003). However, the leaves of small plants can easily reach thermal limits due to overheating caused by decoupling from the atmospheric temperature (Körner and Paulsen 2004; Löffler et al. 2006). For *K. procumbens*, absolute leaf temperature maxima of +50.9°C were recorded, and heat episodes (> 40°C) lasting up to 4.5 h were frequently observed (Neuner and Buchner 2023). Overheating is particularly pronounced on wind‐protected south‐facing slopes above the tree line, where heat loads can become critically high around midday (Neuner et al. 2000; Braun et al. 2002; Buchner and Neuner 2003; Körner and Paulsen 2004; Marcante et al. 2014; Bürli et al. 2021), making heat exposure ecologically important in alpine habitats (Neuner and Buchner 2023). This thermal aspect of North/South solar exposure has received more attention (Bliss 1962; Moser et al. 1977; Körner 1999; Crawford 2008), than the difference in irradiance intensity.

Overheated leaves face a steep gradient of water vapour to the atmosphere. This can lead to critical water losses through transpiration. Stomatal closure is beneficial in such situations as it dramatically reduces water loss. However, water loss continues through incompletely closed stomata and the cuticle, collectively known as leaf minimum conductance (*g*_min_; Schuster et al. 2017; Duursma et al. 2019). Recent studies have highlighted the critical role of *g*_min_ in determining plant water loss during drought, especially under rising atmospheric vapour pressure deficit (VPD). Unlike stomatal conductance, which actively responds to environmental stress, *g*_min_ reflects the cuticular permeability to water vapour when stomata are closed. As VPD increases globally with climate change, this residual water loss becomes a significant factor influencing plant desiccation rates and overall drought survival. In this context, *g*_min_ is now recognised as a key predictor of drought resistance and a limiting factor for residual transpiration (Brodribb et al. 2020; Liang and Ye 2024). Notably, the water permeability of plant cuticles increases exponentially above 35°C (Eamus et al. 2008; Schuster et al. 2016). Leaf temperatures above 35°C are commonly observed in *K. procumbens* (Neuner and Buchner 2023). Further, the cuticle protects against pathogens and reduces damage caused by solar irradiation (Riederer and Schreiber 2001; Riederer and Müller 2006; Heredia‐Guerrero et al. 2018). Its structure and chemical composition can vary between and within plant species and it consists of microlayers that contain different amounts of waxes, cutin, terpenoids, phenolic compounds and carbohydrates (Guzmán et al. 2014; Fernández et al. 2016; Guzmán‐Delgado et al. 2016; Sasani et al. 2021). Among these components, waxes—including epicuticular and intracuticular—consist of long‐chain aliphatics and cyclic triterpenoids, whose physicochemical properties influence water permeability, mechanical protection and adaptation to environmental stress. To improve our state of knowledge on compositional and structural variations in different species and under different environments, microscopic as well as chemical approaches are needed (Fernández et al. 2016). Among the different methods, Raman micro‐spectroscopy provides non‐destructively spatially resolved chemical information in context with the anatomical microstructure. When the monochromatic laser light interacts with molecular bonds in the sample, a small portion of the scattered light is shifted in energy (inelastic scattering), producing a spectrum that reflects the vibrational modes of the chemical components. This makes it especially powerful for mapping in‐situ the distribution of chemical components in different plant tissues (Gierlinger et al. 2012). This technique provided already some insights into the cuticle of different plant species, for example, *Prunus laurocerasus* (Yu et al. 2007), *Arabidopsis* sp. (Bock et al. 2021), *Picea abies* (Sasani et al. 2021), *Dionysia tapetodes* (Bourdon et al. 2021) and tomato (González Moreno et al. 2023). However, apart from these few plant species and organs, plant cuticle diversity remains poorly explored, especially for alpine plant species.

This study focused on the cuticle of *K. procumbens* plants growing naturally under different micro‐environmental conditions at the summit of Mount Patscherkofel (Tyrol, Austria). The micro‐environmental differences found on north‐ and southeast‐facing slopes are hypothesised to force acclimatory adjustments of functional traits (e.g., *g*_min_, photoprotection) within the phenotypic plasticity of cuticle structure and biochemistry. Therefore, we investigated the chemical components and variations in the cuticle structure of leaves grown under two different microclimates by Raman imaging together with water permeability by measuring the minimum diffusive conductance (*g*_min_) at 25°C, as well as possible changes at higher temperatures (38°C, 41°C and 43°C). Understanding the specific cuticle fine‐tuning to cope with specific environmental constraints on north‐ versus southeast‐facing slopes will help to gain knowledge on structural and chemical changes under certain micro‐environments in alpine habitats.

## 2 Materials and Methods

### 2.1 Plant Material and Study Sites

*K*. *procumbens* (L.) Gift, Kron & P.F. Stevens was sampled from two contrasting sites on Mount Patscherkofel, known for its intense foehn winds with up to 150 km∙h^−1^ and more. The first study site (Table 1) was located on the northern slope (*N‐site*), which is densely covered by *K. procumbens* carpets with leaves appearing deep green in colour (Figure S1). The second study site was located at similar elevation on a southeast‐facing slope (*S‐site*), where many but rather scattered and smaller canopies of the species can be found on rocky and gravelly soil. The leaves are not completely green but rather tend to be yellowish (Figure S1). The leathery leaves are about 6 × 2 mm, irregular on the adaxial side, then slightly curled downwards with two longitudinal furrows containing stomata and trichomes (Figure S2). The furrows are parallel to the midvein or midrib (Reisigl and Keller 1987).

**Table 1:** Location, elevation, orientation/azimuth and inclination of the *N‐site* and *S‐site*.

Site	Location	Elevation	Orientation/azimuth	Inclination
*N‐site*	47°12′29″ N/11°27′42″ E	2238 m	N/350°	40°–50°
*S‐site*	47°12′34″ N/11°27′50″ E	2235 m	SE/135°	50°–60°

### 2.2 Microenvironment

At both sites, two identically designed microclimate stations (CR1000, AM25T, Campbell Scientific, Loughborough, UK) were placed within *K. procumbens* carpets. To record short‐term microclimate changes, data were collected at high frequency (1 min intervals) from 1 June 2023 to 15 September 2023. Leaf temperatures (*n* = 12/site) were measured using fine‐wire thermocouple sensors (0.13 mm; TT‐Ti‐36 Omega Inc., Stamford, CT, USA) attached to the abaxial side of the leaves using magnetic leaf clamps (Buchner et al. 2013). The wind speed was measured 10 cm above the ground using ultrasonic‐based anemometers (Windsonic 75, Gill Instruments, Lymington, UK). Photosynthetic photon flux density (PPFD) was recorded using quantum sensors (SKP 215, Skye Instruments, Llandrindod Wells, UK) mounted with their sensing surface oriented horizontally (i.e., facing upward to measure vertically incident radiation).

To determine the effective PPFD for the *N‐site* versus *S‐site* canopy surfaces of *K. procumbens*, the horizontally measured PPFDs were corrected by solar positions and canopy orientations. Solar azimuth and elevation angles were calculated for a nearby site (47.208172° N and 11.460783° E) using the sun tools package in R (Bivand and Luque 2023), with solar positions calculated for each minute during the study period. This solar position information was merged with the PPFD data set to account for changes over time. Because the PPFD sensors recorded values horizontally, a correction factor was applied to adjust the measured values based on the solar elevation angle. For elevation angles > 10°, a correction factor based on the sinus of the solar elevation angle was applied ${\mathrm{PPFD}}_{\mathrm{corrected}}=\frac{{\mathrm{PPFD}}_{\mathrm{measured}}}{\sin(\mathrm{elevation})}$. At low solar elevation angles (< 10°), small changes in elevation caused large corrections, leading to unrealistic spikes. To address this, no correction was applied below 10°, as irradiation at these angles likely reflects diffuse rather than direct sunlight.

Canopy surface orientation effects were simulated by calculating the scalar product between the solar irradiation vector and the normal vector for two exposures (North: 45° elevation, 350° azimuth; Southeast: 35° elevation, 135° azimuth). The scalar values determined effective irradiation for corrected PPFD, and daily maxima were plotted seasonally to show temporal patterns.

### 2.3 Leaf Minimum Diffusive Conductance

The leaf minimum diffusive conductance (*g*_min_) for *K. procumbens* leaves was determined using the mass loss of detached leaves (MLD) technique (Pisek and Berger 1938; Cape and Percy 1996). Leaves were repeatedly weighed during bench drying. After stomatal closure, the initial high water loss decreased rapidly to a constant low mass loss, from which *g*_min_ was calculated using the VPD of the ambient air (Cape and Percy 1996). The mass loss between the two measurements corresponds to the amount of water that transpired. Relating the mass loss to the leaf area provides the current transpiration rate (E). The diffusive conductance (g) for water vapour can be easily calculated from E if the VPD is known, which is the difference between the molar water vapour concentration inside the leaf (close to 100%) and that of the ambient air. Both can be calculated from relative humidity, leaf temperature and air pressure (von Willert 1995). Although *g*_min_ is commonly interpreted as residual conductance when the stomata are closed (Kerstiens 1996), and often interpreted as cuticular conductance (Duursma et al. 2019), recent studies have shown that incomplete stomatal closure may still contribute to *g*_min_, depending on species and conditions (Machado et al. 2021). For our intraspecific comparisons similar stomatal responses after leaf detachment are assumed. In *K. procumbens* stomata are exclusively located in two furrows at the abaxial leaf side and are additionally obscured by a dense mat of non‐glandular trichomes (see Figure S2). The opening gap of the furrows amounts only ~8% of the total leaf surface area, that is, 92% is covered by an epidermal layer without stomata. Removing the trichomes to get a clear view of the stomata was unsuccessful. Therefore, in this species measurement of stomatal openness and traits (e.g., density, size) was not feasible. Under these assumptions, any observed differences in *g*_min_ can be implicitly attributed to variations in cuticular conductance.

Twigs of various lengths (5–20 cm) were collected, placed in plastic bags with moist paper towels, and stored in the dark at 4°C–8°C until the experiments. Small twigs (2–4 cm; *N‐* and *S‐site*: *n* = 36) with 15–45 leaves were randomly selected. The cut stem ends were sealed with warm candle wax (90°C) to prevent water loss. The twigs were weighed (BA 210 S, Sartorius, Göttingen, Germany) and placed on a nylon net (mesh size 5 × 5 mm) for repeated measurements. Simultaneously, the ambient temperature, relative humidity (DK‐RF400, Drießen & Kern, Bramstedt, Germany) and air pressure (GFS 3000, Walz, Effeltrich, Germany) were measured to calculate the absolute humidity of the ambient air and the saturated air within the leaf mesophyll. This process was repeated until diffusive conductance slightly decreased over several measurement intervals, and *g*_min_ was calculated as the mean (Figure S3). Leaves were then separated from the twig, scanned and the projected leaf area was calculated (Image J 1.53t, National Institutes of Health, USA).

The temperature response and heat effect on *g*_min_ were determined by performing *g*_min_ measurements at different exposure temperatures of 25°C, 38°C, 41°C and 43°C. These temperatures were chosen to be sublethal and to occur regularly at natural growing sites during clear summer days (Neuner and Buchner 2023). The temperature treatment during bench drying was performed in the cooling compartment of a switched‐off freezer, with the original lid replaced by a transparent Plexiglas lid (thickness: 3 cm). The internal temperature was kept constant at the selected treatment temperature by two built‐in fan heaters (12 V/100 W, Tru Components C‐MZ45‐FJ120‐12V‐ZK, Conrad Electronics, Wels, Austria), which were controlled by an automatic control system (HTTS8, see Buchner et al. 2013). To the *g*_min_ versus temperature data a general exponential function (1) was fitted (OrginPro 2020, OrginLab Corporation, Northampton, MA, USA).

(1) $$g_{\min(T)}=g_{\min(0^{{}^{\circ}}C)}+A∙\text{exp}\left(R_{0}∙T\right)$$

*g*_min(*T*)_: *g*_min_ at a specific temperature *T* > 0°C; [mmol m^−2^ ∙ s^−1^]

*g*_min(0°C)_: *g*_min_ at 0°C; [mmol m^−2^ ∙ s^−1^]

A: Functional parameter mainly affecting the magnitude of the response of *g*_min_ to increasing temperature.

*R*_0_: Functional parameter mainly affecting the position of the inflection point of the curve along the temperature axis; [°C^−1^].

*T*: Actual temperature; [°C].

### 2.4 Confocal Raman Microscopy and Spectral Analysis

**Figure 1:**
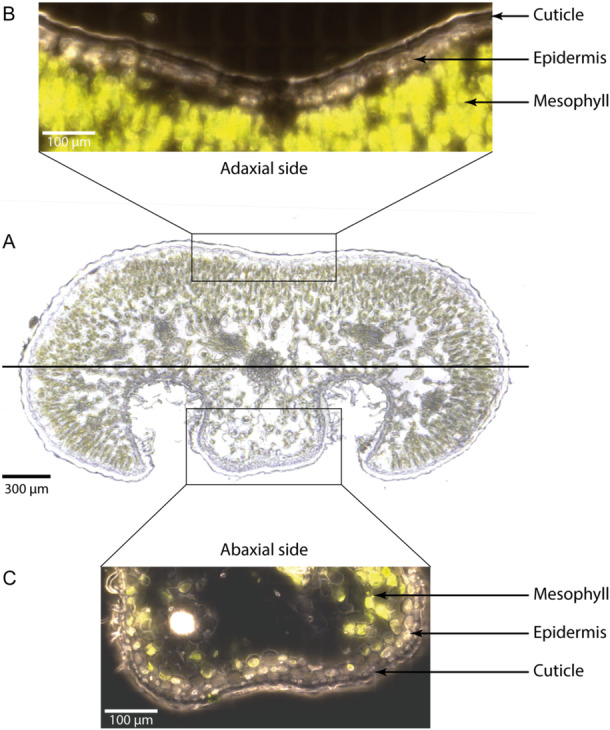
Light microscopic overview stitching image of a *K. procumbens* leaf cross‐section (14 µm thickness, 20× objective, A). Details of the adaxial (B) and abaxial (C) leaf side (100× oil immersion objectives (Nikon, NA = 1.25)) distinguishing cuticle, epidermis and mesophyll cells.

In July 2023, small twigs (< 10 cm in length) of *K. procumbens* were collected from the *N‐* and *S‐site* plots. The twigs were immediately placed in small sealable plastic tubes and transported to the laboratory in a cool bag. They were placed in a freezer at −20°C and then cut using a cryo‐microtome. Raman microscopy is a technique that allows the investigation of the molecular composition of samples, bridging chemical and structural data and gives best results on high‐quality microsections, or microtome cut surfaces (Gierlinger 2018; Mateu et al. 2020). Thus, using a cryo‐microtome (CM 3050 S, Leica Biosystems Nussloch GmbH, Germany), 14–20 µm thick cross‐sections were cut from *K. procumbens* leaves, mounted on a standard microscope slide together with a drop of distilled water, and covered with a microscope coverslip (22 × 22 mm, 170 ± 5 μm, Marienfeld, Germany). To prevent water evaporation and sample movement during the Raman experiments, the samples were sealed with nail polish. Microscopic pictures and hyperspectral data sets were acquired using a confocal Raman microscope (alpha300, WITec, Ulm, Germany) equipped with a piezo scanner. First, an overview stitching image of the whole cross‐section was acquired with the 20× air objective (NA = 0.4, Carl Zeiss, Germany) (Figure 1A). Then, selected regions of interest were stitched with the 100× oil immersion objective (NA = 1.4, Carl Zeiss, Germany) to visualise details and verify section quality before Raman imaging (Figure 1B,C). Hyperspectral data sets were acquired using a linear polarised laser (*λ*_ex_ = 785 laser, WITec, Germany), a wavelength optimised spectrometer (UHTS 30; WITec, Germany) and CCD camera (DU401A‐BV, Andor, UK). Spectra acquisition was controlled using WITec 6.0 control software (WITec, Germany) and a spectrum was taken every 0.3 µm with an integration time of 0.1 s and a laser power of 80 mW.

Prior to generating Raman images, the spectra were cropped (120–1810 cm^−1^ wavenumber range), cosmic rays were removed and baseline‐corrected using WITec Project Plus 6.0. Images of the whole cuticle were obtained by integrating the 1452 cm^−1^ band. Based on these images, the average spectra were extracted from the entire cuticle and its thickness was measured. Additionally, true component analysis (TCA) was performed to retrieve the most chemically different spectra (components) and their distribution (Morel and Gierlinger 2023). Up to six different components were tested, and the three most relevant ‘true’ components were selected and highlighted with different colours, while excluding noise‐related components.

For each chemically distinct region, a representative average Raman spectrum was generated by aggregating the pixel spectra with the highest spectral similarity. The distinguished spectral components may correspond to different molecules or the same (similar) molecules, but mixed in different concentrations.

### 2.5 Scanning Electron Microscopy

Six leaves of *K. procumbens* (three from each site) were cut transversally by using a common razor blade and dried in a lyophilisation chamber for ~2 h before being placed in a desiccator containing silica gel, which was essential to prevent rehydration of the sample. Each section was then placed on a sample holder and gold sputter coated (Leica EM SCD005, Germany). Detailed surface scans of cross‐sections were acquired from the two contrasting sites using an Apreo VS Scanning Electron Microscope (Thermo Scientific, The Netherlands) (SEM) in OptiPlan mode (T1, 10 kV, 0.1 nA) and the cuticle thickness measured. The SEM data were selected for further statistical analysis using R Studio software (R Studio, R Core Team, 2023).

### 2.6 Statistical Analysis

A two‐way ANOVA test was used to simultaneously assess the effect of the two grouping variables (exposure site and leaf side) on cuticle thickness. When significant differences were found, a multiple pairwise comparison between the group means was done using Tukey's test (Tukey HSD, Tukey Honest Significant Differences). Homogeneity of variances and assumption of normality were checked using Levene's test and the Shapiro–Wilk test on the residuals. Thickness average values are presented as means ± standard deviation (SD).

The distribution of the *g*_min_ data was checked for normality by the Kolmogorov–Smirnov test. Thereafter, significant differences in *g*_min_ at a specific temperature between the two sites (*N* vs. *S‐site*) were tested by *t*‐test or by Mann–Whitney *U* test (if normality was not given). The Kruskal–Wallis test procedure was applied for determining significant differences of *g*_min_ from the same site (*N* or *S‐site*) but at different exposure temperatures (IBM SPSS Statistics 27, Armonk, NY, USA).

## 3 Results

### 3.1 Microenvironment at the *N*‐ Versus *S‐Site*

**Figure 2:**
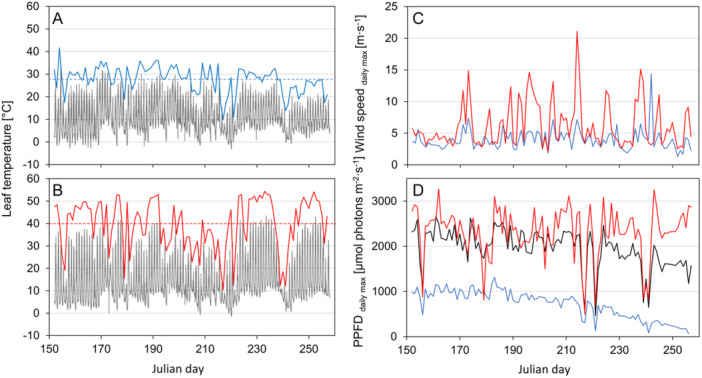
Microclimate of *K. procumbens* at the *N‐* versus *S‐site*. Mean leaf temperatures (*n* = 12, black lines) and daily maximum leaf temperatures at the *N‐site* (A, blue line) and the *S‐site* (B, red line). Horizontal lines represent the mean daily maximum leaf temperatures of the related sites. (C) Daily maximum wind speed at the *N‐site* (blue line) and the *S‐site* (red line). (D) Daily maximum photosynthetic photon flux density (PPFD_max_). The black line represents the mean PPFD_max_ measured by the horizontally orientated sensors. Considering the different canopy surface orientations, the simulated effective PPFD_max_ at the *N‐site* (blue line) and *S‐site* (red line) show largely divergent maximum values. Data were recorded from 1 June 2023 to 14 September 2023.

Leaf temperatures were significantly higher at the *S‐site* than at the *N‐site* throughout the observation period (Figure 2A,B). Absolute leaf temperature maxima reached 54.4°C at the *S‐site*, about 13 K higher than at the *N‐site* (41.6°C). Mean leaf temperatures were also elevated at the *S‐site* (13.8°C ± 9.8°C; *p* < 0.001) compared to the *N‐site* (10.3°C ± 6.3°C; *p* < 0.001). In contrast, the *N‐site* experienced more frequent nocturnal frosts (43 nights below 0°C, absolute minimum −5.0°C) than the *S‐site* (14 nights, absolute minimum –3.1°C).

Wind conditions reflected the prevailing southerly airflow, with both mean and maximum daily wind speeds were significantly higher at the *S‐site* (1.05 m ∙ s^−1^; 6.28 m ∙ s^−1^ respectively, *p* < 0.001) than at the *N‐site* (0.89 m ∙ s^−1^; 3.97 m ∙ s^−1^ respectively, *p* < 0.001), with an absolute maximum of 21.10 m ∙ s^−1^ and 14.40 m ∙ s^−1^, respectively (Figure 2C).

While horizontally measured mean daily maximum PPFD did not differ between sites (*N‐site*: 2020 ± 448 Mol photons m^−2^ ∙ s^−1^; *S‐site*: 1976 ± 437 µMol photons m^−^^2^ ∙ s^−1^; *p* = 0.466), the simulated effective daily maximum PPFD (PPFD_max_) was more than three times higher at the *S‐site* (2319 ± 546 µMol photons m^−2^ ∙ s^−1^; Figure 2D, red line) than at the *N‐site* (709 ± 311 µMol photons m^−2^ ∙ s^−1^; Figure 2D, blue line). At the *N‐site*, PPFD_max_ gradually decreased from mid‐July onwards, which was also seen a little later in the horizontal records. This was not the case at the *S‐site*, where the PPFD_max_ remained unchanged until the end of the measurement period.

### 3.2 Minimum Diffusive Conductance (*g*_min_)

**Figure 3:**
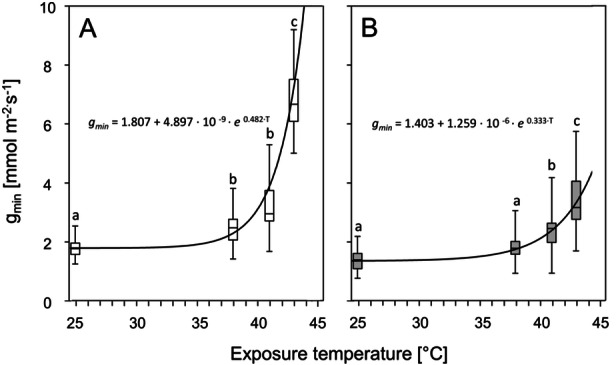
Minimum diffusive conductance (*g*_min_) of *K. procumbens* leaves from two contrasting orientated slopes just below the summit of Mt. Patscherkofel (2248 m a.s.l.). White boxplots (A) are related to leaves from a north‐oriented plot (*N‐site*), and grey boxplots (B) are related to a south‐east‐oriented plot (*S‐site*). Data show the results of drying tests at different ambient temperatures (25°C, 38°C, 41°C and 43°C). The increase in *g*_min_ with increasing temperature follows a standard exponential function for both sites. Significant differences between *g*_min_ at different ambient temperatures are indicated by different lowercase letters, always referring to the relevant sub‐figure (A or B), (Kruskal–Wallis test, *p* < 0.05).

The overall temperature response of *g*_min_ was exponential at both sites (Figure 3). Starting from the low values (mean ± SD) measured at 25°C (*N‐site*: 1.78 ± 0.32 mmol m^−2^ ∙ s^−1^, *S‐site*: 1.39 ± 0.46 mmol m^−2^ ∙ s^−1^), *g*_min_ was already increased at 38°C (*N‐site*: 2.58 ± 0.75 mmol m^−2^ ∙ s^−1^, *S‐site*: 1.86 ± 0.46 mmol m^−2^ ∙ s^−1^). At 43°C at the *N‐site g*_min_ was four times higher (6.91 ± 1.20 mmol m^−2^ ∙ s^−1^) (Figure 3A), while at the *S‐site g*_min_ was only doubled (3.53 ± 1.21 mmol m^−2^ ∙ s^−1^) (Figure 3B), (*p* < 0.001).

In addition to the strong temperature dependence of *g*_min_, there was also a significant difference between the two sites, as *g*_min_ from the *N‐site* at each exposure temperature (25°C, 38°C, 41°C and 43°C) was significantly higher (*p* < 0.001) compared to the *S‐site*.

### 3.3 Cuticle Thickness

**Figure 4:**
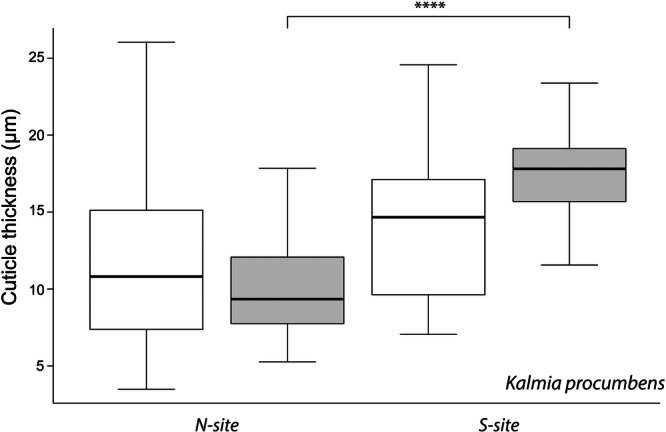
Cuticle thickness of *K. procumbens* from abaxial (white) and adaxial (grey) leaf sides grown at different exposition (*N*‐ vs. *S*‐*site*). Cuticle thickness was measured from different leaves at each leaf side and exposition site by using a scanning electron microscope (SEM) with high‐resolution images to clearly discriminate the cuticle on the adaxial and abaxial side. Adaxial sides from both expositions differ significantly (**** *p* < 0.001). Statistical results are shown in Table S3.

Cuticles were significantly thicker at the *S‐site* than at the *N‐site* (15.9 ± 4.2 µm and 10.9 ± 4.4 µm, respectively; *F* = 87.03, *p* < 0.001, Table S1, Figure 4). While leaf side alone had no effect (*p* = 0.277, Tables S1 and S2), there was a significant interaction between exposure and leaf side (*p* < 0.001, Table S1). Adaxial cuticles from *S‐site* leaves were the thickest overall, significantly exceeding both abaxial side of the same exposure (mean difference = 3.17 µm, *p* < 0.001) and the adaxial cuticles from the *N‐site* (mean difference = 7.52 µm, *p* < 0.001) (Table S3). Conversely, adaxial cuticles from the *N‐site* were thinner than abaxial cuticles from the *S‐site* (mean difference = −4.35 µm, *p* < 0.001) (Table 3). Variability was generally greater on abaxial surfaces. Overall, exposure exerted a dominant effect on cuticle thickness, with *S‐site* adaxial cuticles showing the highest median value (Figure 4).

### 3.4 Cuticle Microchemistry Based on Raman Imaging

**Figure 5:**
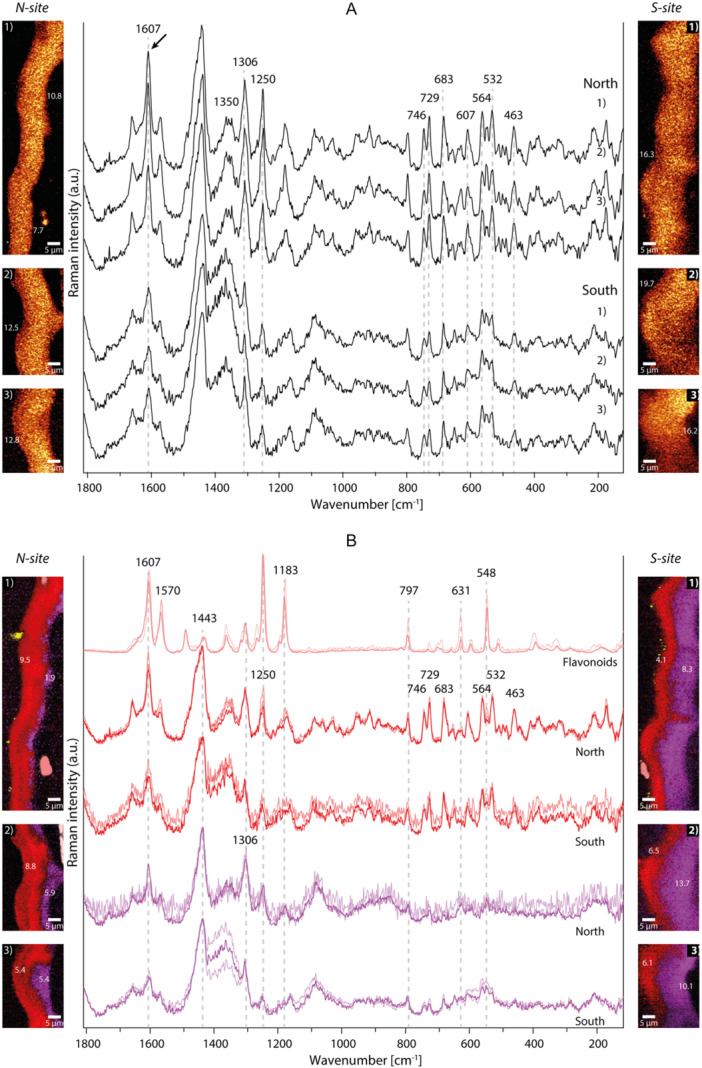
Raman imaging and spectra of *K. procumbens* adaxial cuticles from *N‐* and *S‐site*. (A) Integrating the cutin band at 1452 cm^−1^ shows the entire cuticle and allows to extract average spectra of the cuticle. *N‐site* cuticles were thinner in all three exemplarily shown data sets (1–3) and their average spectra showed higher signals for bands assigned to aromatics (North, black arrow). *S‐site* cuticles were thicker (1–3), with lower aromatic bands in the average spectra (South). (B) True component analysis (TCA) separates cuticle layers and reveals spectral changes within the cuticle. The cuticle has a two‐layered structure: an outer layer rich in aromatic signals (B, red layers and spectra) and thicker in the *N‐site*, and an inner layer with less aromatics (B, magenta layers and spectra) and thicker in the *S‐site*. Flavonoids were detected atop the cuticle in *S‐site* leaves, below cuticle in the *N‐site* (B, salmon layers and spectra).

**Figure 6:**
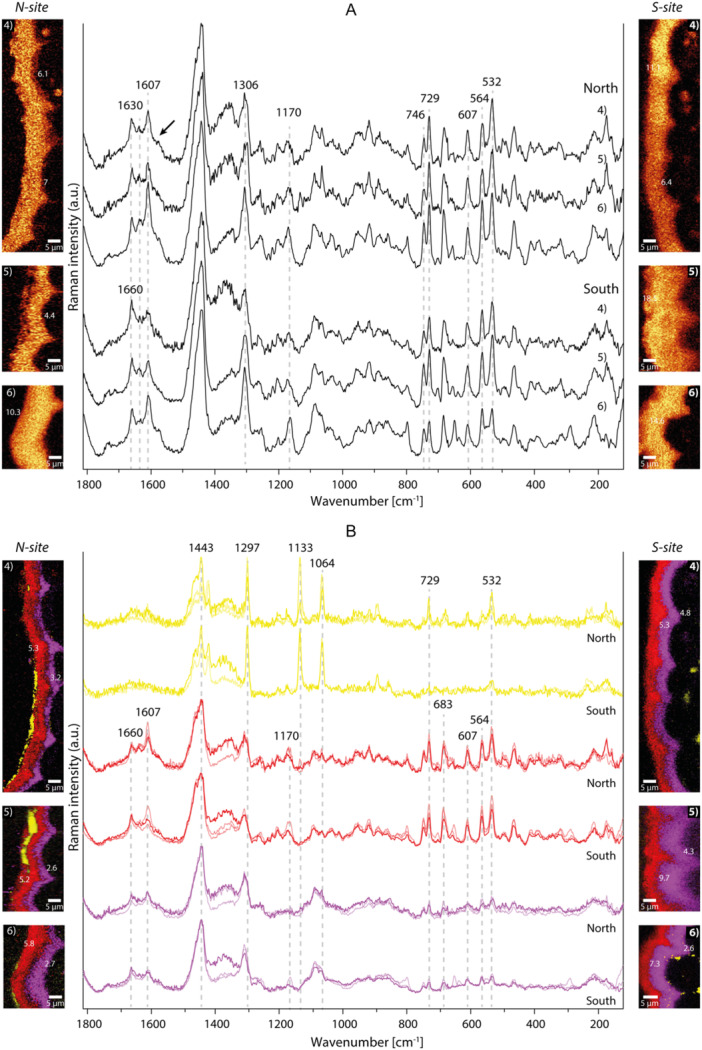
Raman imaging and spectra of *K. procumbens* abaxial cuticles from *N‐* and *S‐site*. (A) 1452 cm^−1^ band integration shows cuticle distribution and related average spectra; (B) combined images (TCA) and related spectra show different layers and composition in cuticle. Average spectra show less aromatic composition, and no flavonoids (A, black arrow) but more cinnamic acids. Long‐chain alkanes were detected externally on leaves from the *N‐site* and below cuticle in leaves from the *S‐site* (B, yellow layers and spectra). Tendencies in thickness and chemical composition followed the results of the adaxial side (B, red and magenta, layers and spectra).

Exemplarily, we show 12 Raman image scans: 6 from the adaxial side and 6 from the abaxial side. Band integration images of the CH‐ (cutin) band at 1452 cm^−1^ reflected the entire cuticle and were used to extract the average spectra (Figures 5, 6), while TCA analysis resolved the chemically different layers, shown in Raman abundance maps of the distinguished components and their average spectra (Figures 5, 6).

#### 3.4.1 Adaxial Side

The average Raman spectra of the *N‐site* showed a pronounced aromatic band at 1607 cm^−1^ (Figure 5A, *N‐site* 1, 2, 3, black arrow), while in the thicker *S‐site* cuticles, the aromatic band intensities were lower (Figure 5A, *S‐site* 1, 2, 3). Based on TCA two layers were distinguished (Figure 5B, red, magenta) as well as accumulation of components on top and inside (Figure 5B, salmon). The most intense Raman spectra came from these accumulations in some epidermal regions (Figure 5B, *N‐site* 1, 2, salmon) or in one case on the external surface (Figure 5B, *S‐site* 1, salmon). The peak at 1607 cm^−1^ points to aromatic ring stretching vibrations, and the peaks at 1570 and 1250 cm^−1^ are distinctive of flavonoids (Krysa et al. 2022). The adjacent layers reflected some of the aromatic bands (e.g., 797, 1183, 1250 or 1607 cm^−1^), confirming cuticle impregnation with flavonoids. However, flavonoids may not be exclusive because of additional bands that point to anthocyanins (e.g., 631, 1630 cm^−1^) (Merlin et al. 1985, 1993). The outer layer was found to be consistently thicker in leaves from the *N‐site*, with a thickness ranging from 5.4 to 9.5 µm (Figure 5B, *N‐site* 1, 2, 3, red). In addition to flavonoids and anthocyanins, these outer layer spectra included many smaller bands in the 800–500 cm^−1^ region (Figure 5B, red spectra). Most of these sharp bands are representative of triterpenoids (463, 532, 564, 683, 729 and 746 cm^−1^), and the band at 746 cm^−1^ points specifically to ursolic acid and/or oleanolic acid (Brody et al. 2002; Yu et al. 2007). The strong bands at 1443 and 1306 cm^−1^ can be attributed to cutin (Colthup and Daly 1990; Prats Mateu et al. 2016) and were found in both outer and inner layers. The inner layer was consistently thicker in leaves from the *S‐site* (thickness range 8.30–13.70 µm, Figure 5B, *S‐site* 1, 2, 3, magenta layer) and with less aromatic contributions.

#### 3.4.2 Abaxial Side

In the average Raman spectra from the abaxial side the intensity of the bands assigned to aromatic ring stretching (1607 cm^−1^) decreased (Figure 6A). The flavonoid peaks at 1570 and 1250 cm^−1^ were not found, but the two bands at 1630 and 1170 cm^−1^ indicated cinnamic acid (e.g., *p*‐coumaric acid) on the abaxial side (Swislocka et al. 2012) (Figure 6A). By TCA analysis again two to three different layers were distinguished (Figure 6B, yellow, red, magenta). The outermost layers of the *N‐site* cuticle were composed of epicuticular waxes, as evidenced by the peaks at 1064, 1133 and 1297 cm^−1^, characteristic of long‐chain alkane crystalline structures (Czamara et al. 2015) (Figure 6B, yellow). Epicuticular waxes are mixed with triterpenoids, as their bands are detected in the region between 800 and 500 cm^−1^ (Figure 6B, yellow spectra). These alkanes were distinguished below the cuticle at the *S‐site* (Figure 6B, *S‐site* 1, 3) with less intense triterpenoid bands (Figure 6B, *S‐site*, yellow spectra). The cuticle layer with high aromatic and triterpenoid content (Figure 6B, red spectra) was again thicker in the *N‐site* cuticles (5.20–5.80 µm, Figure 6B, red), whereas the inner layer tended to be thicker (5.30–9.70 µm, Figure 6B, magenta) and having lower aromatic and triterpenoid band intensities (Figure 6B, magenta spectra) in the *S‐site* cuticles.

## 4 Discussion

**Figure 7:**
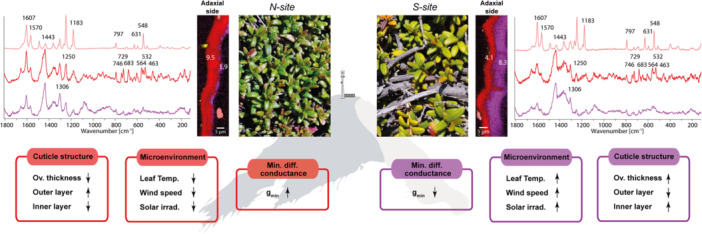
Schematic summary of cuticle structure, microclimate, biochemistry and minimum diffusive conductance (*g*_min_) in *K. procumbens* leaves on the north (*N‐site*, left) and south (*S‐site*, right) slopes. Leaves from the *N‐site* are visibly greener, while those from the *S‐site* exhibit more yellowish colour. Exemplarily Raman images of adaxial cuticles reveal two distinct layers, with the outer one being thicker in *N‐site* leaves and thinner in *S‐site* leaves, where the inner layer becomes thicker. Microenvironment at the *N‐site* is characterised by lower leaf temperature, reduced wind speed, and decreased solar irradiation but higher *g*_min_ compared to the *S‐site*.

*K. procumbens* leaves growing naturally at north‐ versus southeast‐facing slopes but similar elevation experienced different micro‐environments and showed significant differences in cuticle structure, biochemistry and water permeability (Figure 7).

### 4.1 Micro‐Environmental Differences

At the *S‐site K. procumbens* plants exhibited much higher daily maximum leaf temperatures and were exposed to three times higher solar irradiation and stronger winds. The thermal difference between the contrasting sites, as shown in Figure 2, corroborates earlier findings (Moser et al. 1977; Körner 1999; Braun et al. 2002). Small alpine plants, especially cushion and prostrate species, exhibit increased rates of physiological processes at warmer plant body temperatures (Körner 1999) but are occasionally prone to heat stress (Buchner et al. 2013, 2017; Neuner and Buchner 2023). At the *S‐site* daily maximum leaf temperatures of *K. procumbens* regularly exceeded the range of critical heat thresholds around 50°C, but were still below the maximum heat tolerance of 56.4°C (LT_50_) that the species can reach after heat hardening (Buchner and Neuner 2003). At the *N‐site*, heat stress was absent as leaf temperature maxima were ~13 K lower than that at the *S‐site*.

While the thermal aspect of slope exposure driven by solar input has been addressed earlier (Cantlon 1953; Körner 1999; Bennie et al. 2008; Crawford 2008), these studies did not explicitly assess differences in solar irradiation, namely PPFD. Our simulation at the *S‐site* revealed a mean PPFD level of 2319 µMol photons m^−2^ ∙ s^−1^, which is more than three times higher than at the *N‐site* (709 µMol photons m^−2^ ∙ s^−1^). Still, PPFD at the *N‐site* is close to the light saturation point of photosynthesis of *K. procumbens* canopies (Grabherr and Cernusca 1977). At the *S‐site*, PPFD is far beyond saturating light intensities for photosynthesis. Under environmental stress, such as the measured heat load at the *S‐site*, these PPFD intensities are highly excessive, bearing the potential to cause photooxidative damage. Under excessive irradiation, plants often respond with chlorophyll degradation (Blumthaler et al. 1997; Karadar et al. 2018) as seen in the *S‐site* leaves with their yellowish appearance (Figure S1).

Both higher wind speed and overheating, as measured at the *S‐site*, are important environmental factors affecting the water vapour loss of the *K. procumbens* canopies (Grabherr and Cernusca 1977; Körner and Hiltbrunner 2021). The increased wind speed enhances convective heat transfer and VPD around the leaves, which, combined with elevated temperatures, leads to increased water vapour loss. Stomata are highly sensitive to VPD and close upon recognition of critical values (Monteith 1995), even when the leaves are in full sunlight. When stomata close, the water permeability of the cuticle comes into play and thus *g*_min_ plays an important role for plant water use during heatwaves (Kala et al. 2016). Our results show that *g*_min_ of *S‐site* leaves is significantly lower than that of *N‐site* leaves and the difference further increases under heat exposure (> 38°C). Duursma et al. (2019) reviewed the acclimation potential of *g*_min_ to drought, temperature, humidity, leaf age and altitude. They concluded that intraspecific plasticity is high, while a study on *Citrus aurantium* leaves grown under different conditions found no significant effects on water permeability (Geyer and Schönherr 1990). We found in heat stressed *K. procumbens* leaves from the *S‐site*, significantly lower *g*_min_, that appears as a significant phenotypic functional adjustment relevant for survival.

### 4.2 Cuticle Properties in Relation to the Environment

One structural change observed between the cuticles grown under the different micro‐environmental differences was the thicker *K. procumbens* cuticle at the *S‐site* compared to the *N‐site*. In the literature irradiation intensity and angle of incidence have been shown to affect cuticle thickness. Leaves exposed to low irradiance often receive more diffuse irradiation (Knohl and Baldocchi 2008; Košvancová‐Zitová et al. 2009). Diffuse irradiation is reflected more efficiently from the leaf surface because it penetrates the cuticle less than direct irradiation due to its high angle of incidence (Cui et al. 1991; Vogelmann and Gorton 2014; Durand et al. 2021), resulting in the formation of a less thick cuticle than in leaves exposed to direct irradiation (Vogelmann and Gorton 2014). The angle of irradiation incidence at the *N‐site* is flatter than at the *S‐site* and it is suggested that differences in cuticle thickness correlate with differences in the irradiation situation.

Based on Raman imaging the cuticle of *K. procumbens* from the *N‐site*, was particularly rich in flavonoids (Figures 5, 6, 7). On the adaxial leaf surface flavonoids accumulated within the cuticle as well as below the epidermal cell wall, while the abaxial side was rich in phenolic acids. Especially, the outermost layer was thicker in cuticles from *N‐site* and the spectra showed beside the cutin signals, strong bands attributed to triterpenoids and flavonoids (e.g., 1607, 1570 or 1251 cm^−1^, Figure 5B, red layer) (Brody et al. 2002). High light exposure combined with UV‐B irradiation are known to significantly increase both carotenoid and phenolic levels (McElroy et al. 2006; Biswas et al. 2020). However, flavonoids are also known to play a significant role in plant–pathogen interactions (Ziv et al. 2018; Patil et al. 2024), which have been reported in *K. procumbens*, particularly in areas with low wind speed (Petrini 1987) like on the *N‐site*.

The second layer beneath was thicker in *S‐site* cuticles with Raman signals attributed to cutin and triterpenoids. Triterpenoid signals appeared more prominent in abaxial cuticles, but it has to be considered that the higher flavonoid signals in the spectra from the adaxial surfaces may partly mask the triterpenoid signal. As in all *K. procumbens* cuticles and layers triterpenoids were found, these components are considered as typical for the alpine azalea cuticle. Due to their high melting points and resistance to thermal stresses, triterpenoids can enhance plant adaptation in extreme habitats (Casado and Heredia 2001). They have been identified within the cuticle matrix of a desert plant, where they improve the physical properties of waxes and reduce heat damage (Schuster et al. 2016). In *K. procumbens* the presence of triterpenoids in the cuticle could help mitigate heat stress measured in the current study and recently reported for the species (Neuner and Buchner 2023).

The outermost cuticle is typically covered by a hydrophobic layer predominantly composed of long‐chain aliphatic compounds, known as epicuticular waxes. Epicuticular waxes serve as a protective barrier against environmental stressors, including UV irradiation and water loss (Riederer and Schreiber 2001; Yeats and Rose 2013; Berhin et al. 2022). On the adaxial side, both *N‐* and *S‐sites* leaves exhibited an irregular presence of epicuticular waxes, which might be attributed to the limited spatial resolution (~300 nm) of the Raman imaging approach and/or artefacts due to sample preparation (microsectioning). Epicuticular waxes are often deposited as crystals, which might get lost during sample preparations.

### 4.3 Functional Differences

*K. procumbens* cuticle differed structurally and chemically between *N‐* and *S‐site* leaves and the question arises if this phenotypic plasticity may be linked to the change in leaf minimum conductance (*g*_min_)? While both sites exhibited an exponential increase in *g*_min_ with rising temperatures, *N‐site* leaves lost significantly and progressively more water above 38°C. In previously studied species, cuticle water permeability showed also a slight increase at temperatures between 15°C and 35°C, followed by a species‐specific, pronounced increase above 32°C–47°C (Riederer and Schreiber 2001; Riederer 2006; Eamus et al. 2008; Neuner and Buchner 2023; Wang et al. 2024, 2025). Given the predicted increase in heatwaves associated with climate change (Kala et al. 2016), this finding seems significant, particularly as dramatic increases in water loss can be expected in such situations. Nevertheless, our data demonstrate a distinct functional phenotypic plasticity of *g*_min_ in response to heat exposure under natural microclimatic gradients, expanding on earlier findings (Duursma et al. 2019), which focused on acclimation to long‐term warming.

A common perception is that thicker cuticles lower *g*_min_. However, studies have shown that thin cuticles can be just as effective at preventing water loss as thicker ones (Becker et al. 1986). Cuticular water permeability from 61 species was found not to be correlated with cuticle thickness or wax coverage (Riederer and Schreiber 2001). Moreover, *g*_min_ from 30 tropical savanna species correlated more strongly with stomatal traits than cuticle thickness (Machado et al. 2021). Yet, if looking at the same species grown at different elevation, a relationship between thinner cuticles from pine needles grown at the alpine timberline and higher water permeability, was reported (Bueno et al. 2022). In our study, the lower *g*_min_ values in the *S‐site* leaves coincided with thicker cuticles, and suggested a possible association, although a direct causal link cannot be inferred. The thinner cuticle of the *N‐site* might partly explain the higher water permeability. However, an effect of differently closed stomata can be ruled out. Stomata in *K. procumbens* are restricted to the furrows on the abaxial side and densely covered by hairs (see Figure S2), which makes assessment of stomatal openness and traits not feasible. In experiments with *K. procumbens* leaves whose furrows had been sealed with superglue, similar *g*_min_ values to those obtained in the current study using the MLD technique were obtained using an infrared gas analyser (unpublished data) suggesting full stomatal closure in our experiments.

The significant higher water permeability at higher temperature may be related to the higher flavonoid content in the *N‐site* cuticles. Currently, we do not know exactly the molecular composition and the reaction of the flavonoids upon heat treatment. In case they are hydrophilic flavonoids (with more hydroxyl groups) they could lose water during heat treatment, resulting in reorganisation (isomerisation, crystallisation) and formation of more pores for water diffusion. Thus, a biotic adaptation of higher flavonoid content to defend fungi, might on the other hand increase water permeability. However, this interpretation remains speculative and needs further testing, as the specific physicochemical behaviour of these compounds upon heating is not yet fully understood.

Our findings are supported by Grünhofer et al. (2022), who showed that even a > 10‐fold increase in cuticular waxes in *Populus* x *canescens* leaves did not reduce *g*_min_. This decoupling of waxes and permeability emphasises that functional changes in cuticular water loss are not necessarily driven by wax quantity or chain length, but also by qualitative chemical traits or heat‐induced reorganisation. It seems that phenotypic plasticity of the cuticle, especially under environmental stress, must be interpreted beyond mere thickness, stomatal traits or wax amount.

## 5 Conclusion

The results of our study shed new light on the phenotypic acclimation potential of plants in alpine environments, under similar macroenvironmental conditions, but slope induced contrasting microenvironments. We recorded higher leaf temperatures, stronger winds and exceptionally high levels of solar irradiance at the *S‐site* compared to the *N‐site*. At the heat‐prone *S‐site*, *K. procumbens* showed chlorophyll degradation and developed thicker cuticles that better protect against water loss, particularly under heat. The *N‐site* cuticles seemed to adapt to biotic stresses and invest in flavonoid impregnation. Our results not only elucidate the mechanisms by which plants cope with certain environmental factors but also highlight the critical role of plant secondary metabolites and their distribution across the leaf surface in plant acclimation to environmental and biotic stresses.

## Conflicts of Interest

The authors declare no conflicts of interest.

## Supporting information


**Supporting information 1:** Site‐specific leaf colouration of *K. procumbens* leaves. The two study sites were located at similar elevations but on differently oriented slopes just below the summit of Mount Patscherkofel (2248 m a.s.l.). While the leaves at the *N‐site* (2238 m a.s.l, left picture) are usually fresh green in colour, the leaves from the *S‐site* (2235 m a.s.l., right picture) often show a yellowish colour and sometimes exhibit heat damage (arrows). White circles: Ripe and open capsules. Date: 15 September 2023. **Supporting information 2:** Scanning Electron Microscopy (SEM) pictures of *K. procumbens* leaf surfaces showing (A) the adaxial side, (B) the abaxial sides, and (C) the cross‐section. Regions of interest are highlighted in different colours corresponding to areas selected for higher‐magnification imaging. **Supporting information 3:** Determination of the minimum diffusive conductance. Typical course of the diffusive conductance for water (g) of *K. procumbens* leaves during controlled dehydration at an ambient temperature of 38°C. It takes approx. 3.5 h until the stomata of the well‐saturated leaves are maximally closed. Thereafter, g remains at a constant low value (g_min_) for 17 h. The subsequent downward slope of the curve (open symbols) is because the leaves have already dried out considerably and therefore no longer reflect the actual diffusive conductance. **Suppl. Tab.1:** Outcomes from the applied factorial ANOVA test on cuticle thickness across adaxial and abaxial leaf sides from *N*‐ and *S‐site*. **Suppl. Tab. 2:** Overall average thickness for adaxial and abaxial sides across both *N*‐ and *S‐site* including mean, standard deviation (SD), sample size (N), standard error (SE), 95% confidence intervals bound (CI lower, CI upper). **Suppl. Tab.3:** Post‐hoc comparisons (Tukey HSD) for cuticle thickness between exposition sites and leaf sides, and their interaction effects, including mean difference in cuticle thickness (Diff.), 95% confidence intervals bound (CI lower, CI upper), p‐value (p).

## Data Availability

Data will be made available on request.
