# Supplementary material for: Phenotypic Cuticle Plasticity at High Elevation: Is Microstructure and Microchemistry Related to Water Permeability?
Source: Plant Cell Environ. 2025 Dec 18;49(3):1778–92. doi: 10.1111/pce.70344 (PMC12873512; doi:10.1111/pce.70344)
Supplement: Supplementary file 1 — Supporting information 1: Site‐specific leaf colouration of K. procumbens leaves. The two study sites were located at similar elevations but on differently oriented slopes just below the summit of Mount Patscherkofel (2248 m a.s.l.). While the leaves at the N‐site (2238 m a.s.l, left picture) are usually fresh green in colour, the leaves from the S‐site (2235 m a.s.l., right picture) often show a yellowish colour and sometimes exhibit heat damage (arrows). White circles: Ripe and open capsules. Date: 15 September 2023. Supporting information 2: Scanning Electron Microscopy (SEM) pictures of K. procumbens leaf surfaces showing (A) the adaxial side, (B) the abaxial sides, and (C) the cross‐section. Regions of interest are highlighted in different colours corresponding to areas selected for higher‐magnification imaging. Supporting information 3: Determination of the minimum diffusive conductance. Typical course of the diffusive conductance for water (g) of K. procumbens leaves during controlled dehydration at an ambient temperature of 38°C. It takes approx. 3.5 h until the stomata of the well‐saturated leaves are maximally closed. Thereafter, g remains at a constant low value (gmin) for 17 h. The subsequent downward slope of the curve (open symbols) is because the leaves have already dried out considerably and therefore no longer reflect the actual diffusive conductance. Suppl. Tab.1: Outcomes from the applied factorial ANOVA test on cuticle thickness across adaxial and abaxial leaf sides from N‐ and S‐site. Suppl. Tab. 2: Overall average thickness for adaxial and abaxial sides across both N‐ and S‐site including mean, standard deviation (SD), sample size (N), standard error (SE), 95% confidence intervals bound (CI lower, CI upper). Suppl. Tab.3: Post‐hoc comparisons (Tukey HSD) for cuticle thickness between exposition sites and leaf sides, and their interaction effects, including mean difference in cuticle thickness (Diff.), 95% confidence intervals bound (CI lower, C [file PCE-49-1778-s001.docx]

**Supporting information 1** Site-specific leaf colouration of *K.* *procumbens* leaves. The two study sites were located at similar elevations but on differently oriented slopes just below the summit of Mount Patscherkofel (2248 m a.s.l.). While the leaves at the *N-site* (2238 m a.s.l, left picture) are usually fresh green in colour, the leaves from the *S-site* (2235 m a.s.l., right picture) often show a yellowish colour and sometimes exhibit heat damage (arrows). White circles: Ripe and open capsules. Date: 15 September 2023.

***
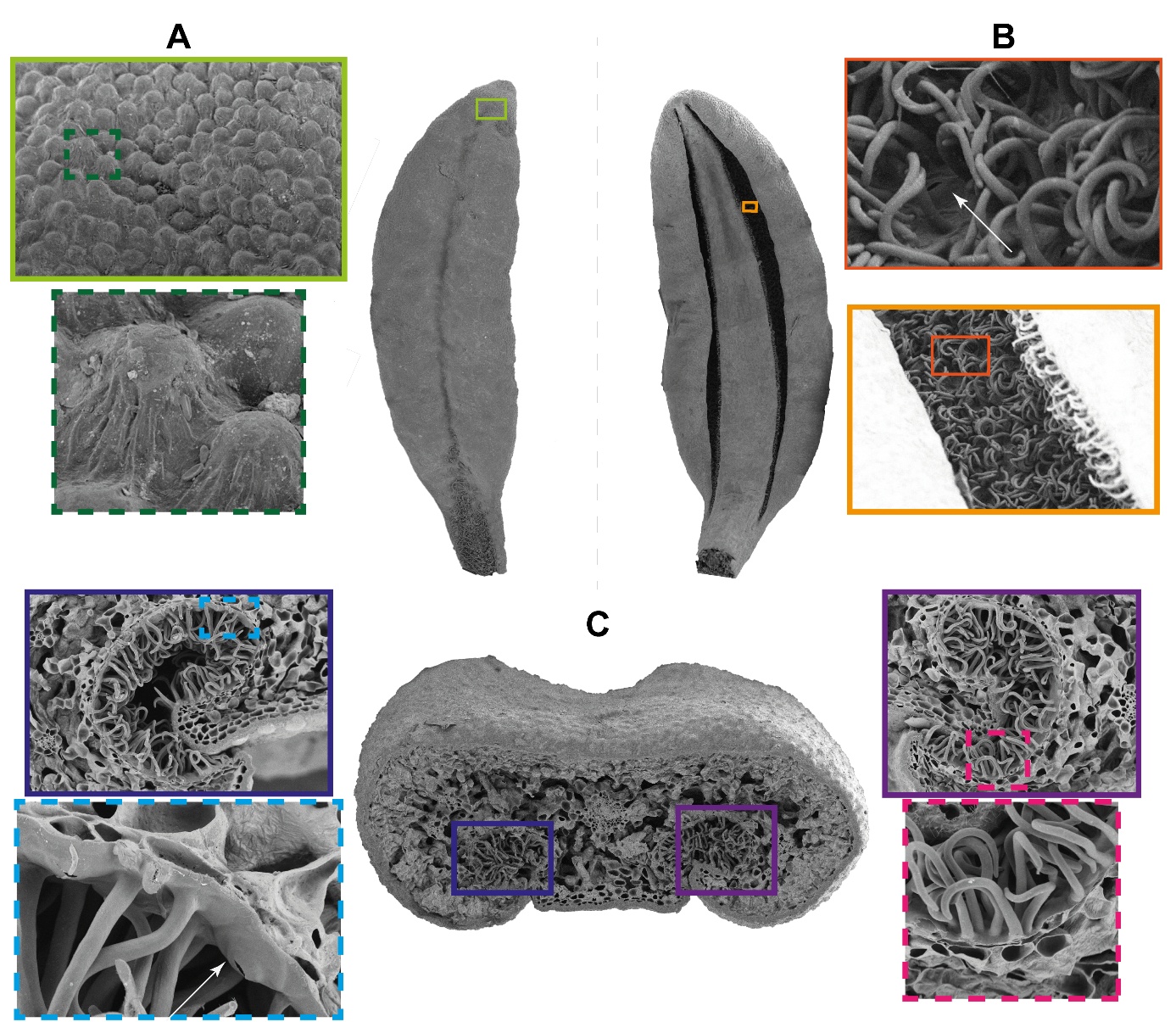
***

**Supporting information 2** Scanning Electron Microscopy (SEM) pictures of K. procumbens leaf surfaces showing (A) the adaxial side, (B) the abaxial sides, and (C) the cross-section. Regions of interest are highlighted in different colours corresponding to areas selected for higher-magnification imaging.


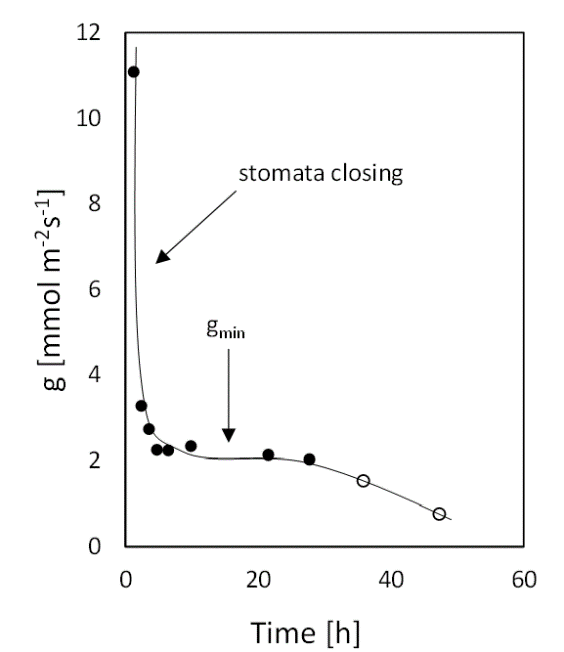
**Suppl. Tab. 1** Outcomes from the applied factorial ANOVA test on cuticle thickness across adaxial and abaxial leaf sides from N- and S-site.

**Supporting information 3** Determination of the minimum diffusive conductance. Typical course of the diffusive conductance for water (g) of *K.* *procumbens* leaves during controlled dehydration at an ambient temperature of 38°C. It takes approx. 3.5 h until the stomata of the well-saturated leaves are maximally closed. Thereafter, g remains at a constant low value (g_min_) for 17 h. The subsequent downward slope of the curve (open symbols) is because the leaves have already dried out considerably and therefore no longer reflect the actual diffusive conductance.

|  | Df | Sum Sq | Mean Sq | F value | Pr (>F) |
| --- | --- | --- | --- | --- | --- |
| Exposition | 1 | 1552 | 1552.20 | 87.03 | < 0.001 |
| Leaf side | 1 | 21 | 21.20 | 1.19 | 0.28 |
| Eposition~  Leaf side | 1 | 431 | 431 | 24.17 | < 0.001 |
| Residuals | 252 | 4494 | 17.80 |  |  |

**Suppl. Tab. 2** Overall average thickness for adaxial and abaxial sides across both N- and S-site including mean, standard deviation (SD), sample size (N), standard error (SE), 95% confidence intervals bound (CI lower, CI upper).

|  | Mean | SD | N | SE | CI lower | CI upper |
| --- | --- | --- | --- | --- | --- | --- |
| Abaxial | 13.20 | 5.32 | 128 | 0.470 | 12.30 | 14.10 |
| Adaxial | 13.80 | 4.77 | 128 | 0.421 | 12.90 | 14.60 |

**Suppl. Tab. 3** Post-hoc comparisons (Tukey HSD) for cuticle thickness between exposition sites and leaf sides, and their interaction effects, including mean difference in cuticle thickness (Diff.), 95% confidence intervals bound (CI lower, CI upper), p-value (p).

|  | Diff. | lwr | upr | p |
| --- | --- | --- | --- | --- |
| S-site –  N-site | 4.92 | 3.88 | 5.96 | < 0.001 |
| Adaxial –  Abaxial | 0.57 | -0.46 | 1.61 | 0.28 |
| S:Abaxial – N:Abaxial | 2.33 | 0.40 | 4.26 | 0.01 |
| N:Abaxial – N:Adaxial | -2.01 | -3.95 | -0.09 | 0.04 |
| S:Adaxial – N:Abaxial | 5.50 | 3.57 | 7.43 | <0.001 |
| N:Adaxial – S:Abaxial | -4.35 | -6.28 | -2.42 | <0.001 |
| S:Adaxial – S:Abaxial | 3.17 | 1.24 | 5.10 | <0.001 |
| S:Adaxial – N:Adaxial | 7.52 | 5.59 | 9.45 | <0.001 |
